# The value of motherhood and psychological distress among infertile women: The mediating role of coping strategies

**DOI:** 10.3389/fpubh.2023.1024438

**Published:** 2023-02-02

**Authors:** Florentina Larisa Foti, Adina Karner-Huţuleac, Alexandra Maftei

**Affiliations:** Faculty of Psychology and Education Sciences, Alexandru Ioan Cuza University, Iaşi, Romania

**Keywords:** infertility, women, psychological distress, coping strategies, motherhood

## Abstract

**Introduction:**

The present study investigated psychological distress and coping strategies among infertile women during the COVID-19 pandemic using a multi-dimensional model of infertility-related stress. We explored the associations between individual (i.e., age) and situational characteristics related to infertility (i.e., duration of infertility, cause of infertility, number of lost pregnancies, and assisted reproductive techniques [ART] status), and perceived-infertility-related factors (i.e., the perceived importance of motherhood).

**Methods:**

Our total sample consisted of 193 women aged 20 to 46 (M= 33.23, SD = 4.58), out of which 102 were undergoing ART procedures (*M* = 33.60, *SD* = 4.23), and 91 were not (*M*= 32.81, *SD* = 4.94). Participants filled in questionnaires measuring psychological distress, coping strategies, and the importance of motherhood.

**Results:**

Correlation analyses suggested that the importance of motherhood was positively associated with psychological distress and negative coping strategies. Mediation analysis results indicated that both in the overall sample and in the sample of women undergoing ART procedures, the negative self-perception fully mediated the link between the importance of motherhood on psychological distress. In the non-ART sample, we found a significant mediation effect of denial on the link between the importance of motherhood and psychological distress.

**Discussion:**

We discuss the theoretical and practical implications of the present findings, focusing on the mental health-related consequences of the social stigma of infertility heightened by the pressure of parenthood.

## Introduction

The problem of infertility [i.e., the inability to conceive after 12 months of unprotected intercourse or after 6 months if a woman is 35 years or older] (1, 2) has been a constant concern over the years among researchers due to its complex implications at various personal and social levels (3, 4). It is generally estimated that infertility affects around 25 million people in the European Union alone (5). The Romanian Association for Human Reproduction (6) suggested that around 17% of the examined fertile population (4,680 participants) were in an infertility situation, and 38% of these couples sought infertility treatment.

Treating infertility is a common practice, although it is influenced by factors such as genetics, social expectations about motherhood, and public opinions and attitudes toward infertility and assisted reproductive techniques (ART), such as *in-vitro* fertilization (IVF) procedures (7). However, the choice of treatment relies on the exact cause of infertility, with approaches ranging from ovulation induction using a variety of medicines to more technically advanced procedures like IVF (8). Ovulatory disorders, for example, are among the most common medical causes of reduced reproduction, and a considerable percentage of couples worldwide are affected. Blocked fallopian tubes, endometriosis, or uterine anomalies are among the conditions that can reflect female infertility.

### Infertility and psychological distress

The link between stress and infertility has generally been studied using a bidirectional approach, i.e., exploring the impact of stress on fertility and the impact of infertility on stress and overall psychological wellbeing. If the answers to the question “*Does stress affect fertility*?” might seem contradictory, according to the previous studies (9–12), the answer to the question “*Does infertility affect stress?*” seems significantly more straightforward (13).

Even if it is not a life-threatening diagnosis, infertility is still a highly stressful experience. Infertility has been linked to decreased marital wellbeing (14) and sexual functioning (15). Additionally, infertility can lead to feelings of failure, anxiety, depression, remorse, grief, and guilt (16, 17). This diagnosis can also negatively affect self-esteem (18, 19), with low levels leading to anxiety and depression (20).

Stress, anxiety, and depression are specific forms of psychological distress, generally more common among females than males (21). Previous research addressing the link between psychological distress and infertility (20, 22–30) generally highlighted the positive link between these variables. A systematic review and meta-analysis study concluded that the prevalence of depression was higher among infertile women than in the general population of a given country (25).

It was also suggested that infertile women experience significantly higher levels of depression, anxiety, and stress compared to their spouses (18, 22, 31), especially since women are considered complete only when they become mothers (26). Furthermore, depression is often followed by social isolation and low self-esteem because of gender discrimination (16) and the fact that motherhood is considered a highly desirable achievement (32). At the same time, Kiani et al. (33) suggested that infertile women report high anxiety levels due to the unpredictability of fertility treatment outcomes and the long-term nature of ART procedures.

Furthermore, among infertile women, higher depression was positively associated with low self-esteem (20, 34), high levels of shame (27, 35), stress (36), social concern, sexual concern and maternal relationship stress (37). Finally, the importance of parenthood was indirectly associated with depression, throughout the association of experiential avoidance, as a coping strategy and the perceived impact of infertility (38).

### ART, age, and duration of infertility

Regarding the impact of assisted reproductive technologies, most previous studies suggested that repeated ART might be linked to higher distress in anxiety and depression (39, 40), though the findings in this area are mixed (41). Furthermore, previous studies (42–44) also suggested that the number of lost pregnancies and the duration of infertility (45–48) might be significantly related to infertile women's psychological distress. A review study (42) concluded that infertility and perinatal loss are associated with major depressive disorder, anxiety, posttraumatic stress disorder, complicated grief, marital discord, and low quality of life. The authors also highlighted the importance of treating anxiety and depression because infertility and perinatal loss may be caused or perpetuated by these symptoms. Along with high levels of depression and anxiety following the loss, infertile women also experience feelings of shame, self-blame, social awkwardness, fear, profound loss and grief, feelings of personal responsibility for what had happened, injustice or lack of fairness and inadequacy (44).

Regarding the duration of infertility, previous studies suggested that as the duration of infertility increases, depression may also increase (39, 46–48). Some studies have concluded that the long duration of infertility is positively associated with stress and anxiety (45). However, other studies suggested that the duration of infertility might not be directly associated with anxiety (39, 49) but rather mediated by the importance of motherhood (41).

Finally, when it comes to age, studies suggested that it might be negatively associated with infertile women's anxiety (34) and might not correlate with depression (34, 50). On the other hand, other studies found a positive correlation between age and distress, anxiety, and depression (37, 51). Thus, the results in this area are mixed and call for further research.

### A multi-dimensional model of infertility-related stress

Based on the Transactional Theory of Stress (TTS); (52), Zurlo et al. (49) proposed a multi-dimensional model of infertility-related stress. The TTS emphasizes that several risk (individual and situational characteristics) and protective factors (e.g., coping strategies) must be considered when predicting psychological health. Based on this conceptual approach, Zurlo et al. (49) expanded it and added a series of demographic characteristics and coping strategies as part of individual characteristics. In addition, infertility-related parameters (i.e., type of diagnosis and duration of infertility), perceived sources of stress in infertility (i.e., social concern; couple's relationship concern; the need for parenthood; rejection of childfree lifestyle), and the perceived dyadic adjustment dimensions (i.e., dyadic consensus; affectional expression; dyadic cohesion; and dyadic satisfaction) were also added as situational characteristics.

The model proposed by Zurlo et al. (49) aimed to explore the predictive role of these factors when discussing psychological distress (i.e., anxiety and depression) among both partners of couples undergoing infertility treatments. In the present study, we used this theoretical approach, which we extended by adding some specific variables related to ART status and the number of lost pregnancies.

### Coping with infertility

Coping strategies, considered individual characteristics, include cognitive, emotional, and behavioral efforts to manage internal or external sources of stress and are the major factor in adaptational outcomes (52, 53). Folkman and Moskowitz (54) classify coping strategies into four categories: problem-focused (behavioral strategies that address the problem that causes distress), emotion-focused (aimed to reduce negative emotions associated with the problem), meaning-focused (cognitive strategies that help the person to understand the problem), support seeking (reducing stress by reaching out to the community for emotional support).

These strategies are the subject of various studies related to the level of psychological distress among infertile individuals (49, 55), which investigated them as a mediator (56) and as a moderator (49) in the relationship between the sources and the outcomes of psychological distress. On the other hand, the relationship between coping strategies and anxiety and depression has been shown to be moderated by the duration of infertility (57) and mediated by the quality of life (58).

Among infertile women, active coping strategies (e.g., problem-solving, seeking social support) have been associated with low levels of distress (49, 59), while passive strategies (e.g., avoidance, denial, disengagement, social withdrawal) have been associated with increased levels of distress (35, 60). Regarding the potential gender differences, previous studies suggested that females, compared to males, seem to resort more often to *social support* (49, 61), which is associated both positively with anxiety (62), as well as negatively (22), in addition to being negatively related to infertility-related stress (63).

Coping strategies can be subject to cultural specificities (64), which can also influence the acceptance or denial of an infertility diagnosis and the option and adherence to treatment (65, 66). Furthermore, in countries with a collectivist culture, as is also the case in Romania, where family and religion are mainstream values (67), turning to divinity in the event of an illness is a strategy that many people might use to reduce the level of associated stress (68). For example, some studies have shown that women facing infertility turn to *religious coping* (69), which might help them reduce their stress, anxiety, and depression (70, 71). However, the literature also distinguishes between positive religious coping (which refers to the perceived help/support that the divinity can provide) and negative coping (infertility is considered a divine punishment). In the case of negative religious coping, these strategies seem to have increased infertility-related psychological distress (69).

Cousineau and Domar (72) suggested that one's *self-perception* can be affected when the only goal is motherhood, which might be hard to achieve. Previous studies have concluded that the inability to have a child leads to numerous psychological problems, including associated stress, anxiety, and negative self-perception (64), and social support might decrease them (73). Karaca and Unsal (74) concluded that infertility harms self-perception due to the perceived social pressure and infertility-related stigma, and similar findings were reported by Coşkuner Potur et al. (75).

*Preoccupation with infertility-related thoughts* can become obsessive, affecting the daily activities of women who want to become mothers and fail (74). Moreover, as the years pass and the number of failed treatments increases, the constant thinking about infertility [e.g., about the effectiveness of treatments, causes of infertility, efforts to find ways to avoid explanations to family/friends, uncertainty about the future – *who* they would be and *how* would their life will look like if they cannot conceive; (76)] becomes a common coping strategy in infertile couples (77). Additionally, interacting with potentially stressful stimuli (e.g., seeing a pregnant woman) triggers significantly more intrusive thoughts in females than in males (78).

Finally, infertility-related stress might also occur because of society-related and cultural factors and norms, i.e., the idea that having a child is mandatory. Thus, the related social pressure might lead to emotional imbalance and the need for social withdrawal or keep the diagnosis a secret (13, 79).

### The importance of motherhood

Though voluntary childlessness (i.e., the option of fertile couples not to have children) - as an alternative to parenthood - appears to be gaining popularity globally (80), in many parts of the world (especially in countries with pronatalist policies), motherhood is associated with a higher social status (81), since children are an important source of social desirability (82). At the same time, cultural norms and beliefs favor reproduction, resulting in a predominantly negative opinion of childless women (83). People believe that a life without children cannot be fully satisfying (84). Adults who choose not to have children are stigmatized, viewed as aberrant, egocentric, or lacking in a feeling of responsibility (85). Women without children are typically seen more perceived more negatively than those having children (86). In Romania, according to the Barometer of Public Opinion (87), 83% of Romanian adults ranked family and children as the most important component of their lives, followed by religion.

Other studies concluded that motherhood represents a woman's most important goal and source of ultimate satisfaction (32, 88), but this aspect is also associated with high anxiety (49). Furthermore, studies investigating infertility suggest that infertile women struggle to cope with the stigma and powerlessness associated with not fulfilling this prescribed social norm [i.e., motherhood; (89)].

The implications of the importance of motherhood in the mental health of infertile women have been the subject of several studies (38, 90, 91). Most of them suggested that socially-prescribed motherhood (which is also associated with high social pressure) seems to predict infertility-related distress (91). It has also been suggested that the desire to become a parent is associated with one's wellbeing, life satisfaction, stress, and depression (90, 92). On the other hand, some studies suggested that infertility and, consequently, the inability to fulfill this role doesn't directly lead to depression but somewhat indirectly, through feelings of inadequacy and unfulfillment (38, 93).

Furthermore, according to recent data (94, 95), Romania is one of the most religious countries in Europe [with 86.6% Orthodox population, according to the (96)], and this also reflects on the importance of motherhood and the various pathways to parenthood, in general (2). Furthermore, when it comes to motherhood and the general perception regarding the pathway to becoming a parent in the case of women affected by infertility, according to Maftei and Holman (2), adoption seems to be the most preferred option among Romanian women, followed by IVF and surrogacy.

### The present study

**Figure 1:**
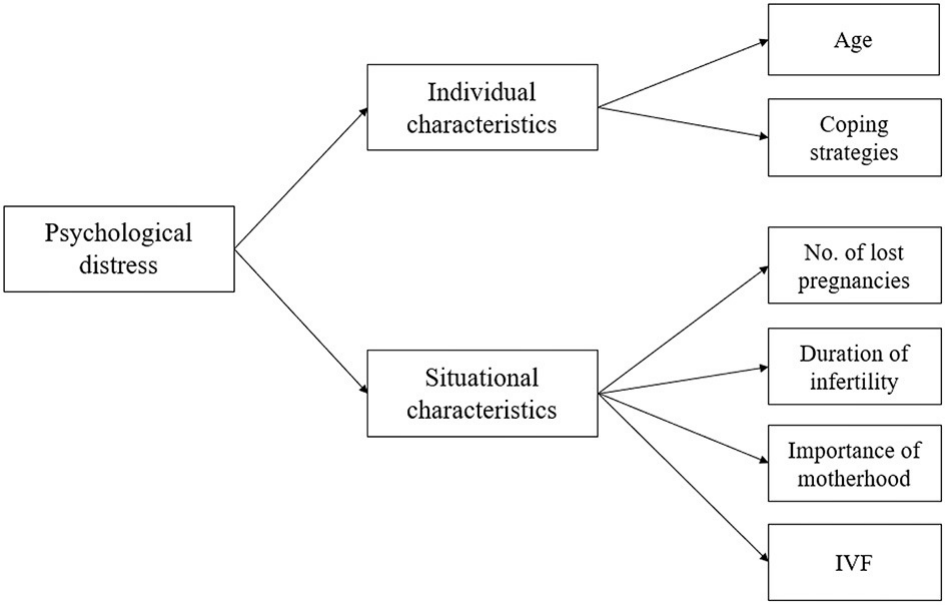
The proposed conceptual framework, based on Zurlo et al. (49).

The present study was built on the multi-dimensional model of infertility-related stress proposed by Zurlo et al. (49). We aimed to explore the associations between *individual* characteristics such as sociodemographic factors (i.e., age) and coping strategies, and *situational* characteristics related to infertility-related parameters, i.e., duration of infertility, cause of infertility, number of lost pregnancies, ART status (i.e., whether the participants were undergoing ART procedures at the time of the research), and perceived-infertility-related factors, such as the perceived importance of motherhood (see Figure 1).

The novelty of our study lies in (1) the addition of the variable concerning the number of lost pregnancies as an infertility-related parameter, in line with previous studies (42, 44, 47) that suggested its importance when examining the emotional outcomes related to infertility, (2) the addition of the ART-status variable, since previous studies documented its important role, as well, when discussing infertility-related distress (39, 40), (3) the moment of our research, i.e., during the COVID-19 pandemic.

Based on this theoretical model and the previous related literature, the general assumptions were the following: *H1*. There would be significant differences concerning psychological distress and maladaptive coping strategies, depending on women's ART status (i.e., participants who were undergoing ART procedures at the time of the research and women who were not). More specifically, according to previous literature, we assumed that women who were not undergoing ART would report higher psychological distress and more maladaptive coping strategies (41); *H2*. Regardless of the ART status, the importance of motherhood would be positively associated with maladaptive coping strategies. Next, as we added to Zurlo's model (49) the number of lost pregnancies as an infertility-related parameter, we assumed that *H3*. The number of lost pregnancies would be significantly associated with infertile women's psychological distress and maladaptive coping strategies; more specifically, we assumed that the higher the number of lost pregnancies, the higher the psychological distress and maladaptive coping strategies. Finally, we also assumed that *H4*. Maladaptive coping strategies would have a significant indirect effect on the link between the importance of motherhood and participants' psychological distress.

## Method

### Participants and procedure

One hundred ninety-three women aged 20 to 46 (*M* = 33.23, *SD* = 4.58) participated in our study. Most of them were married (86%), employed (around 80%), and had a Bachelor's or a Master's degree. The demographic scale we used also included details related to the number of children and residency (rural or urban). The inclusion criteria that we used were the following: women aged at least 18, with an infertility diagnosis, who wanted children. Though the medical diagnosis of female infertility was a condition to participate in our study, we also asked participants to state the perceived cause of their infertility. Additionally, the participants also reported the number of lost pregnancies and whether they used assisted reproductive techniques (intracytoplasmatic sperm injection, artificial insemination, gamete intrafallopian transfer, IVF). All these details are presented in Table 1.

**Table 1:** Sample characteristics (*N* = 193).

**Variable**	***N***	**%**
**Residency**		
Rural	25	13.0
Urban	168	87.0
**Marital status**		
Married	166	86.0
In a stable relationship	25	13.0
Single	2	1.0
**Religion**		
Orthodox	175	90.7
Catholic	5	2.6
Atheist	5	2.6
Other	8	4.1
**Children**		
Yes	18	9.3
No	175	90.7
**Highest educational level**		
Elementary school	2	1.0
High school	19	9.8
Post-secondary school	7	3.6
Short-cycle tertiary education	2	1.0
Bachelor's degree	71	36.8
Master's degree	86	44.6
PhD	6	3.1
**Employment status**		
Student	2	1.0
Employed	154	79.8
Employer	9	4.7
Self-employed	14	7.3
Retired	1	0.5
Maternity leave	6	3.1
Unemployed	7	3.6
**Cause of infertility**		
Female	93	48.2
Male	22	11.4
Couple	29	15.0
Idiopathic/unexplained	24	12.4
No diagnose yet	10	5.2
Missing	15	7.8
**Assisted reproductive technology**		
Yes	149	77.2
No	44	22.8
**Duration of infertility (years)**	*M* = 4.04 (*SD* = 3.84)	
**Number of lost pregnancies**	*M* = 0.58 (*SD* = 1.08)	

All participation was voluntary. We used convenience sampling using the snowballing technique. We distributed the participation link *via* specialized online support groups (e.g., Facebook groups associated with the medical clinics that treated infertile women) and, with the help of three physicians (obstetricians who worked in those clinics), the link was also directly distributed to the patients who agreed to be informed of such research opportunities (e.g., *via* e-mail or WhatsApp messages).

The instruments were administered at the end of 2021 (October-December). The participants were informed of the participation requirements, incentives, and their right to withdraw from the study at any time. We also informed all participants that their answers would remain confidential and anonymous and would only be used for the present research. The research protocol was developed following the ethical guidelines from the university with whom the authors are affiliated and the 2013 Helsinki Declaration. The average time needed to answer the items was around 35 min.

### Measures

#### Psychological distress

We measured psychological distress using the 21-item Depression, Anxiety, and Stress Scale [DASS-21, (97)]. We selected this scale because of its documented dependability and efficacy (i.e., a relatively small number of items measuring three psychological dimensions). Using a scale ranging from 0 (did not apply at all) to 3 (very applicable), participants rated the applicability of each item (7 items for each subscale) considering the preceding week. Example items included “I couldn't seem to experience any positive feeling at all” (*depression*); “I was aware of dryness of my mouth” *(anxiety*); and “I found it hard to wind down” (stress). Cronbach's alpha-s for all three scales indicated good reliability, i.e., α = 0.89 for depression, α = 0.90 for anxiety, and α = 0.89 for stress. Higher scores indicated higher depression, anxiety, and stress. In the present study, we also computed an overall score for the scale (i.e., Psychological distress).

#### Coping strategies

We used the Coping Scale for Infertile Women [CSIW; (64)] to explore the coping strategies used by the participants in our sample in dealing with infertility-related problems. The items are scored on a 5-point Likert scale ranging from 1 (strongly disagree) to 5 (strongly agree). The scale comprises ten dimensions, i.e., *Preoccupation with thoughts* (e.g., I have physical problems like insomnia and loss of appetite because of my thoughts), *Spiritual coping* (e.g., I believe that God will reward me for dealing with this problem), *Denial* (e.g., I prefer to talk about this problem), *Social withdrawal* (e.g., I prefer to contact with my relatives less often), *Negative self-perception* (e.g., I feel weak and incomplete), *Hope* (e.g., I am dreaming about children), *Social support seeking* (e.g., I ask a relative or a friend, whom I respect or trust, for advice regarding this problem), *Accept* (e.g., I learn to live with this problem), *Investigating in Self* (e.g., I pay more attention to my appearance compared to the past), and *Spousal relations* (e.g., I am trying to involve my partner in each step of the problem/treatment). In the present study, following internal reliability analyses, we used a 34-item version of the scale, comprising six dimensions, i.e., *Preoccupation with thoughts* (α = 0.88), *Spiritual coping* (α = 0.72), *Negative self-perception* (α = 0.83), *Social withdrawal* (α = 0.80)*, Denial* (α = 0.70), and *Social support seeking* (α = 0.76). A high score indicated that the person uses more of that way of coping.

#### The importance of motherhood

We asked participants to evaluate on a scale ranging from 1 (not at all) to 100 (extremely important) how important it was for them to have (another) child.

We used the International Test Commission (98) cross-cultural adaption approaches before using the instruments (99, 100). First, two independent translators transcribed the instrument from English to Romanian. We examined the two translated versions and assessed the potential ambiguities with a third translator. There were no major contradictions, and the consensus allowed the initial translation scales. We then blindly back-translated the initial tentative translation of the instruments and compared the two back-translated scales to create the final instruments.

## Results

### Overview of the statistical analysis

We first conducted preliminary analyses; then, we computed zero-order correlations among the variables and tested for differences depending on the ART status using independent *T*-tests. Finally, we conducted mediation analyses based on these results.

### Preliminary analyses

We used the IBM SPSS 26 statistical software for the analyses. Data cleaning steps and normality checks were performed preliminary to any analyses. Out of the 201 participants who initially formed our sample, eight were eliminated because they stated they did not want children anymore. We then computed the Skewness and Kurtosis values to assess the normality of the distributions, and all the values were in the 2/-2 limit suggested by George and Mallery (101) (see Table 2 for the descriptive statistics of the main variables).

**Table 2:** Descriptive statistics for the main variables (*N* = 193).

**Variable**	**M**	**SD**	**Min**	**Max**	**Skewness**	**Kurtosis**
Depression	6.96	5.70	0.00	21.00	0.61	−0.64
Anxiety	9.58	5.70	0.00	21.00	0.14	−0.89
Stress	7.22	5.84	0.00	21.00	0.61	−0.76
Psychological distress	23.76	15.84	0	60	0.43	−0.76
Preoccupation with thoughts	21.58	7.77	7.00	35.00	−0.02	−0.96
Negative self-perception	20.36	6.81	6.00	30.00	−0.30	−1.03
Social withdrawal	20.90	6.34	6.00	30.00	−0.36	−0.77
Spousal relation	6.16	3.14	3.00	15.00	1.07	0.43
Denial	19.17	5.35	6.00	30.00	−0.11	−0.52
Spiritual coping	17.76	5.58	6.00	30.00	0.13	−0.57

### Associations among the main variables

Independent *T*-tests further suggested marginally significant differences between the participants who were undergoing ART procedures at the time of the research, and women who were not, concerning psychological distress, *t*_(191)_ = 1.96, *p* = 0.052, preoccupation with thoughts, *t*_(191)_ = 2.48, *p* = 0.01, and spiritual coping, *t*_(191)_ = 2.08, *p* = 0.03, with higher rates (in all cases) among women who were not undergoing ART.

We further computed zero-order correlations to investigate the associations between the research variables and test for potential multicollinearity. Given the *t-*test results concerning the differences between ART and non-ART groups, and for a better understanding of emotional outcomes examined (i.e., psychological distress), we examined these links separately for the overall sample (Table 3A), participants who were involved in the time of the research in any ART procedures (Table 3B), and participants who were not involved at the time of the research such procedures (Table 3C).

**Table 3A:** Zero-order correlations among the main variables—overall sample (*N* = 193).

**Variable**	**1**	**2**	**3**	**4**	**5**	**6**	**7**	**8**	**9**	**10**	**11**	**12**	**13**
1. Depression	1												
2. Anxiety	0.75^**^	1											
3. Stress	0.73^**^	0.82^**^	1										
4. Psychological distress	0.89^**^	0.93^**^	0.92^**^	1									
5. Preoccupation with thoughts	0.53^**^	0.47^**^	0.44^**^	0.53^**^	1								
6. Negative self-perception	0.53^**^	0.45^**^	0.42^**^	0.50^**^	0.74^**^	1							
7. Social withdrawal	0.48^**^	0.39^**^	0.30^**^	0.42^**^	0.60^**^	0.70^**^	1						
8. Spousal relation	−0.24^*^	−0.11	−0.15^*^	−0.18^**^	−0.20^**^	−0.36^**^	−0.31^**^	1					
9. Denial	0.29^**^	0.31^**^	0.27^**^	0.31^**^	0.34^**^	0.44^**^	0.43^**^	−0.19^*^	1				
10. Spiritual coping	0.10	0.13	0.18^*^	0.15^*^	0.26^**^	0.22^**^	0.16^*^	0.14^*^	0.15^*^	1			
11. Importance of motherhood	0.10	0.08	0.16^*^	0.12	0.10	0.17^*^	0.15^*^	−0.14^*^	0.25^**^	0.20^*^	1		
12. Age	−0.00	−0.06	−0.07	−0.05	0.01	−0.00	−0.02	0.01	−0.01	−0.02	−0.11	1	
13. No. of lost pregnancies	−0.00	−0.00	−0.05	−0.02	0.01	0.01	0.00	0.01	−0.03	0.02	0.10	0.14^*^	1
14. Duration of infertility	−0.06	−0.09	−0.06	−0.08	0.13	−0.00	−0.00	0.02	−0.03	−0.00	−0.01	0.25^**^	0.02

^*^p < 0.05; ^**^p < 0.001.

**Table 3B:** Zero-order correlations among the main variables—women undergoing ART (*N* = 102).

**Variable**	**1**	**2**	**3**	**4**	**5**	**6**	**7**	**8**	**9**	**10**	**11**	**12**	**13**
1. Depression	1												
2. Anxiety	0.74^**^	1											
3. Stress	0.69^**^	0.78^**^	1										
4. Psychological distress	0.89^**^	0.92^**^	0.90^**^	1									
5. Preoccupation with thoughts	0.55^**^	0.46^**^	0.40^**^	0.52^**^	1								
6. Negative self-perception	0.54^**^	0.41^**^	0.35^**^	0.48^**^	0.74^**^	1							
7. Social withdrawal	0.48^**^	0.37^**^	0.22^*^	0.40^**^	0.64^**^	0.70^**^	1						
8. Spousal relation	−0.26^*^	−0.08	−0.17	−0.19	−0.15	−0.35^**^	−0.27^*^	1					
9. Denial	0.22^*^	0.30^*^	0.16	0.25^**^	0.19	0.30^**^	0.33^**^	−0.07	1				
10. Spiritual coping	0.14	0.20^*^	0.21^*^	0.20^**^	0.25^**^	0.16	0.14	0.25^*^	0.16	1			
11. Importance of motherhood	0.16	0.04	0.10	0.11	0.13	0.23^*^	0.14	−0.18	0.24^*^	0.12	1		
12. Age	−0.02	−0.04	−0.09	−0.09	−0.05	−0.01	0.00	−0.06	0.08	0.02	0.09	1	
13. No. of lost pregnancies	0.01	−0.05	−0.08	−0.04	−0.07	−0.02	−0.06	0.02	0.15	−0.03	0.15	0.32^**^	1
14. Duration of infertility	−0.09	−0.13	−0.05	−0.10	−0.19	−0.03	0.01	−0.06	0.11	0.00	0.08	0.31^**^	0.10

^*^p < 0.05; ^**^p < 0.001.

**Table 3C:** Zero-order correlations among the main variables — women who are not undergoing ART (*N* = 91).

**Variable**	**1**	**2**	**3**	**4**	**5**	**6**	**7**	**8**	**9**	**10**	**11**	**12**	**13**
1. Depression	1												
2. Anxiety	0.74^**^	1											
3. Stress	0.76^**^	0.84^**^	1										
4. Psychological distress	0.89^**^	0.93^**^	0.94^**^	1									
5. Preoccupation with thoughts	0.49^**^	0.47^**^	0.46^**^	0.51^**^	1								
6. Negative self-perception	0.50^**^	0.48^**^	0.47^**^	0.52^**^	0.73^**^	1							
7. Social withdrawal	0.49^**^	0.41^**^	0.39^**^	0.46^**^	0.59^**^	0.71^**^	1						
8. Spousal relation	−0.22^*^	−0.15	−0.14	−0.18	−0.27^*^	−0.36^**^	−0.37^**^	1					
9. Denial	0.33^**^	0.31^**^	0.34^**^	0.35^**^	0.45^**^	0.57^**^	0.55^**^	−0.33^**^	1				
10. Spiritual coping	0.01	0.02	0.11	0.05	0.24^*^	0.28^**^	0.20	−0.00	0.11	1			
11. Importance of motherhood	0.06	0.14	0.22^*^	0.16	0.11	0.12	0.16	−0.11	0.27^*^	0.33^*^	1		
12. Age	0.05	−0.07	−0.09	−0.04	0.01	0.01	0.01	0.03	−0.06	0.01	−0.34^**^	1	
13. No. of lost pregnancies	−0.00	0.06	−0.01	0.01	0.06	0.00	0.04	−0.05	−0.03	0.00	0.05	0.00	1
14. Duration of infertility	−0.02	−0.04	−0.06	−0.04	−0.05	0.06	−0.00	0.03	−0.05	0.01	−0.13	0.33^**^	−0.06

^*^p < 0.05; ^**^p < 0.001.

In the overall sample, the results suggested significant associations between psychological distress and preoccupation with thoughts (*r* = 0.53, *p* < 0.001), negative self-perception (*r* = 0.50, *p* < 0.001), social withdrawal (*r* = 0.42, *p* < 0.001), spousal relations (*r* = −0.18, *p* = 0.01), denial (*r* = 0.31, *p* < 0.001), and spiritual coping (*r* = 0.15, *p* = 0.03). The importance of motherhood was significantly associated with stress (*r* = 0.16, *p* = 0.02), negative self-perception (*r* = 0.17, *p* = 0.01), social withdrawal (*r* = 0.15, *p* = 0.03), spousal relations (*r* = −0.14, *p* = 0.04), denial (*r* = 0.25, *p* < 0.001), and spiritual coping (*r* = 0.20, *p* < 0.001).

In the sample of participants who were not undergoing ART procedures at the time of the research (*N* = 91), the pattern of relations remained relatively similar. The results suggested significant associations between psychological distress and preoccupation with thoughts (*r* = 0.51, *p* < 0.001), negative self-perception (*r* = 0.52, *p* < 0.001), social withdrawal (*r* = 0.46, *p* < 0.001), and denial (*r* = 0.35, *p* < 0.001). The importance of motherhood was positively associated with stress (*r* = 0.22, *p* = 0.03), denial (*r* = 0.27, *p* = 0.008), and spiritual coping (*r* = 0.33, *p* = 0.001).

In the sample of participants who were undergoing ART procedures at the time of the research (*N* = 102), results suggested significant associations between psychological distress and preoccupation with thoughts (*r* = 0.52, *p* < 0.001), negative self-perception (*r* = 0.48, *p* < 0.001), social withdrawal (*r* = 0.40, *p* < 0.001), denial (*r* = 0.25, *p* < 0.001), and, in addition to the non-ART sample, we also found a significant association with spiritual coping (*r* = 20, *p* = 0.03). The importance of motherhood was positively associated with negative self-perception (*r* = 23, *p* = 0.01) and denial (*r* = 0.24, *p* = 0.01).

### Mediation analyses

Based on the results from the correlation analyses, we further used the SPSS macro program PROCESS – Model 4 (30) [95% confidence interval (CI); 5,000 bootstrapped samples] to explore the potential mediating roles of the coping strategies on the link between the importance of motherhood and psychological distress. We examined these indirect effects separately (overall, ART, and non-ART samples).

*a. The indirect effects of negative self-perception, social withdrawal, spousal relation, denial, and spiritual coping on the link between the importance of motherhood and psychological distress (overall sample, N* = *193)*.

We ran the mediation analyses using all four mediators. The results suggested that the total effect of the importance of motherhood on psychological distress was not significant, *b* = 0.10, *SE* = 0.05, *p* = 0.07, 95% CI [−0.01; 0.2], and neither was the direct effect, *b* = 0.01, *SE* = 0.05, *p* = 0.07, 95% CI [−0.08; 0.12]. The only significant indirect effect was the one of negative-self-perception, *b* = 0.05, *SE* = 0.02, 95% CI [0.01; 0.11]. Thus, women's negative self-perception fully mediated the link between the importance of motherhood on psychological distress.

*b. The indirect effects of negative self-perception and denial on the link between the importance of motherhood and psychological distress (ART sample, N* = *102)*.

We ran the mediation analyses using both mediators. The results suggested that the total effect of the importance of motherhood on psychological distress was not significant, *b* = 0.09, *SE* = 0.08, *p* = 0.25, 95% CI [−0.07; 0.27], and neither was the direct effect, *b* = −0.01, *SE* = 0.07, *p* = 0.08, 95% CI [−0.17; 0.14]. The only significant indirect effect was the one of negative-self-perception, *b* = 0.09, *SE* = 0.05, 95% CI [0.01; 0.23]. Thus, as in the case of the overall sample, women's negative self-perception fully mediated the link between the importance of motherhood on psychological distress.

*c. The indirect effects of denial and spiritual coping on the link between the importance of motherhood and psychological distress (non-ART sample, N* = *91)*.

We ran the mediation analyses using both mediators. The results suggested that the total effect of the importance of motherhood on psychological distress was not significant, *b* = 0.12, *SE* = 0.08, *p* = 0.12, 95% CI [−0.03; 0.29], and neither was the direct effect, *b* = 0.06, *SE* = 0.08, *p* = 0.49, 95% CI [−0.11; 0.23]. The only significant indirect effect was the one of denial, *b* = 0.06, *SE* = 0.03, 95% CI [0.01; 0.14]. Thus, denial fully mediated the link between the importance of motherhood on psychological distress in the sample of women who were not undergoing ART procedures at the moment of research.

## Discussion

The present study aimed to explore the associations between *individual* characteristics and coping strategies, *situational* characteristics related to infertility, and perceived-infertility-related factors, such as the perceived importance of motherhood. Our research was based on the multi-dimensional model of infertility-related stress proposed by Zurlo et al. (49).

In line with previous studies (90), our results suggested that the importance of motherhood was positively associated with psychological distress and coping strategies such as negative self-perception, social withdrawal, denial, and spiritual coping. This particular result might highlight the overwhelming role played by social norms that dictate the need to have children and consider motherhood a moral duty (32, 83, 88). Furthermore, mediation analysis results suggested that both in the overall sample and in the sample of women undergoing ART procedures, the negative self-perception fully mediated the link between the importance of motherhood on psychological distress. In the sample of participants who were not undergoing ART procedures at the time of the study, we found a significant indirect effect between the importance of motherhood on psychological distress. These results seem consistent with previous results suggesting that resorting to dysfunctional coping strategies such as avoidance or denial is usually associated with increased levels of psychological distress (35, 60).

Furthermore, these findings generally underline the similarities between the samples (overall, non-ART, and ART), with significant indirect effects of maladaptive coping strategies on the link between the value of motherhood and infertile women's depression, anxiety, and stress. The implications of these findings are both theoretical and practical: first, they add to the literature on psychological distress among infertile women by providing a comparative perspective between women undergoing ART procedures and those who are not. Second, these results highlight the need for psychological support and intervention strategies addressing maladaptive coping and its link with the value placed on motherhood and different social expectations regarding parenting roles (102).

Next, our results suggested marginally significant differences (which also underlines that we should interpret these results with caution) between the ART vs. non-ART samples concerning psychological distress, preoccupation with thoughts, and spiritual coping, with higher rates among non-ART women. In other words, though some studies suggested that ART procedures can be highly stressful for infertile couples (39, 40), our results seem to align with the ones suggested by Moura-Ramos et al. (41), suggesting that the infertile couples undergoing ART might be less stressed than the infertile couples who are not experiencing these procedures.

The low control over the desire to become a parent (i.e., infertility diagnosis) and the desire to achieve this goal might explain why, in the present study, the importance of motherhood was positively associated with stress, denial, and spiritual coping. Turning to religiosity might be a way to reduce infertility-related stress (68), as the desire for biological parenthood can also lead women to turn to religiosity before deciding to use ART (103). The idea is also supported by our results which suggested that, in the case of non-ART participants, the importance of motherhood was associated with religious coping, while in ART sample this association was not observed.

Our findings also suggested that participants' negative self-perception fully mediated the link between the importance of motherhood on psychological distress, both in the overall and in the ART samples. These results are in line with Cousineau and Domar (72), who suggested that one's self-perception can be affected by the value placed on motherhood. This result highlights the need for multidisciplinary teams (e.g., medical professionals and psychotherapists) to work with infertile women's psychological distress to help them overcome the social stigma of infertility.

Our results also suggested that in the case of the non-ART sample, denial fully mediated the link between the importance of motherhood on psychological distress. These results align with the idea that coping strategies can influence the acceptance or rejection of an infertility diagnosis and the option and adherence to treatment (65, 66). The desire to become a biological mother is so high sometimes that it causes infertile women to postpone turning to ART, hardly accepting the diagnosis. This result can be the basis of future psychoeducation interventions to facilitate decision-making among infertile women or couples and increase their wellbeing.

Regarding the current study, some limitations need to be addressed as well. First, our study is not as generalizable as it could have been because we employed a convenient sample size of participants that was relatively small (104). In subsequent research, it may be beneficial to investigate the correlations between the primary variables of our investigation in more comprehensive and diverse samples. We also used self-reported measurements, which enhanced the possibility of desirable answers. For future studies, it may be beneficial to use various assessment methods, such as experimental procedures, to address this limitation.

Another potential limitation might be related to the time of the study, i.e., the COVID-19 pandemic – at a time when the fear of COVID-19 challenged people worldwide. Thus, our findings should be interpreted cautiously, considering this specific factor in future ecological research perspectives. In this regard, the significant psychological impact of the COVID-19 pandemic on infertile women who had *in-vitro* treatment interrupted or postponed should be considered (105, 106).

Also, we did not assess the cultural particularities, even though we know that these aspects may play a significant part in discussing emotional repercussions connected to infertility. Furthermore, we used a cross-sectional approach, which does not allow us to draw any conclusions related to causal relationships between the variables, a limitation that future studies should address. Also, we focused only on heterosexual women, which might also limit the generalizability of our findings (107).

Also, we did not include several other variables that might account for significant changes in the relationships we examined. Out of these factors, we consider the partner's perspective on infertility, the importance of parenthood in men's lives, as well as men's psychological distress (41), might be considered in future studies that would further explore these links while also examining the role played by the COVID-19 pandemic in this regard (108). Also, in the demographic scale, we measured the perceived cause of infertility. Though our approach was not focused on this factor, future studies might want to account for the potential variabilities concerning this factor, especially in such religious contexts as the Romanian one. Next, while our research attempted to compare rural versus urban populations regarding the primary variables of our study, especially given the religious context, the two groups were too unbalanced for further analyses, an issue that might be addressed in further research. Finally, as we previously highlighted, the influence of cultural and religious factors on the value placed on motherhood and the choice to undergo ART is highly significant. However, the importance of these factors can also be regarded in terms of pregnancy termination decisions, and the perception (i.e., value) of motherhood (109, 110).

Our study was based on the multi-dimensional model of infertility-related stress proposed by Zurlo et al. (49). We added to this model the variables concerning the number of lost pregnancies as an infertility-related parameter and the ART-status variable since previous studies documented its important role when discussing infertility-related distress (39, 40). However, our findings did not suggest a significant link between psychological distress and the number of lost pregnancies. However, the differences we found between the ART and non-ART samples highlight the importance of the ART status when examining infertile women's emotional distress and coping strategies (a research path worth examining in future, more extended studies).

## Conclusion

Infertility is a highly stressful life event with an even more significant impact on women's mental health if motherhood is considered a moral duty and a social imperative (91). At the same time, it might be even more stressful when dealing with unprecedented health crises, such as the COVID-19 pandemic. In addition to the theoretical insight added by our study, the practical implications are related to mental health professionals' awareness of the importance of motherhood on the mental health of infertile women, which may lead to a better therapeutic approach. The inclusion, in therapy, of techniques that can help to accept the unpredictability in reaching the proposed goal (e.g., fertility) and increasing the level of resilience can prevent the clinical pathology associated with infertility (primary prophylaxis) or can prevent complications and the unfavorable evolution of the mental health problems (secondary prevention).

## Data availability statement

The raw data supporting the conclusions of this article will be made available by the authors, without undue reservation.

## Ethics statement

This study's protocol was designed the following ethical requirements specific to the Faculty of Psychology and Educational Sciences, Alexandru Ioan Cuza University (Iasi, Romania) before beginning the study and supervised by Florentina-Larisa Foti. Ethical Approval No. 3058/28.09.2021. The patients/participants provided their written informed consent to participate in this study.

## Author contributions

All authors listed have made a substantial, direct, and intellectual contribution to the work and approved it for publication.
